# Plumbagin attenuates Bleomycin-induced lung fibrosis in mice

**DOI:** 10.1186/s13223-022-00734-7

**Published:** 2022-10-21

**Authors:** Saber Mehdizadeh, Marjan Taherian, Paria Bayati, Kazem Mousavizadeh, Salar Pashangzadeh, Ali Anisian, Nazanin Mojtabavi

**Affiliations:** 1 Department of Immunology, School of Medicine, Iran University of Medical Sciences, Tehran, Iran; 2 Immunology Research Center, Institute of Immunology and Infectious Diseases, University of Medical Sciences, Tehran, Iran; 3 Department of Pharmacology, School of Medicine, Iran University of Medical Sciences, Tehran, Iran; 4 Department of Pathology, Islamic Azad University of Abhar, Abhar, Iran

**Keywords:** Plumbagin, Inflammation, Fibrosis, Bleomycin, Idiopathic pulmonary fibrosis

## Abstract

**Background:**

Idiopathic pulmonary fibrosis (IPF) is a fatal fibrotic lung disease with limited treatment options. Plumbagin (PL) is an herbal extract with diverse pharmacological effects that have been recently used to treat various types of cancer. This study aims to explore the anti-fibrotic effect of PL and possible underlying mechanisms in IPF.

**Methods:**

We used a bleomycin-induced experimental mouse model of lung fibrosis to assess the potential anti-fibrotic effect of PL. Histological analysis of lung tissue samples by H&E and Masson’s trichrome staining and hydroxyproline assay was performed to evaluate the fibrotic alterations. ELISA and real-time quantitative PCR were conducted to determine the amount of tumor necrosis factor-alpha (TNFα), tumor growth factor-beta (TGF-β), connective tissue growth factor (CTGF), and endothelin-1 (ET-1).

**Results:**

Bleomycin exposure induced lung fibrosis, which was indicated by inflammation, collagen deposition, and structural damage. PL remarkably prevented bleomycin-induced lung fibrosis. Furthermore, PL significantly inhibited TNF-α and TGF-β production. PL also diminished the upregulated expression of CTGF and ET-1 induced by bleomycin.

**Conclusion:**

Overall, our findings suggest PL as an anti-fibrotic agent acting via down-regulation of TGF-β/CTGF or ET-1 axis, as well as TNF-α, to improve lung fibrosis.

## Background

Idiopathic pulmonary fibrosis (IPF) is a common, chronic and progressive form of interstitial lung disease [1]. The causes of IPF are still unknown; however, a large body of evidence implicates inflammatory injuries to the alveolar epithelium as an initial event promoting the release of pro-fibrotic growth factor, mainly TGF-β, and generation of the fibroblast and myofibroblast foci, in turn, produce a large amount of extracellular matrix (ECM), such as collagens. Massive ECM deposition in lung parenchyma leads to the destruction of the alveolar architecture, loss of lung function, and eventually respiratory failure and death [2, 3]. IPF is associated with rising prevalence and median survival of 3 years from diagnosis, imposing a significant burden on health care systems [4, 5]. Despite this disease burden, there are no available curative therapies approved by the Food and Drug Administration for IPF.

Although it is widely accepted that a chronic inflammatory process precedes the pathogenesis of IPF, anti-inflammatory therapy was not adequate, and immunosuppressive treatment, including azathioprine and prednisolone, was shown to worsen the outcome [6]. In addition, two available licensed drugs for IPF, pirfenidone, and nintedanib, are often poorly tolerated and display limited efficacy in clinical trials, with only the ability to slow disease progression [7–9]. Therefore, more effective antifibrotic treatments remain an urgent need.

Plumbagin (5-hydroxy-2-methyl-1,4 naphthoquinone or PL), an active quinonoid constituent extracted from the roots of the traditional medicinal herb Plumbago zeylanica L, has been indicated to exhibit diverse biological effects, incorporating anti-inflammation, antioxidant, anti-angiogenesis, induction of apoptosis, and anti-tumorgenesis [10–13]. Owning these pharmacological activities, PL is utilized to treat various types of cancers [14]. Also, PL has been shown to exert potential therapeutic benefits on several chronic diseases [15]. Further, it is demonstrated that PL possesses immunosuppressive and anti-inflammatory properties in central experimental autoimmune encephalomyelitis (EAE) [16]. More recently, PL has been reported to effectively ameliorate liver fibrosis via downregulation of epidermal growth factor receptor (EGFR), STAT3, alpha-smooth muscle actin (α-SMA), ROS-mediated NF-кB signaling pathway, and inhibition of inflammation and collagen production [17–20]. Taken together, we supposed that PL might also play an immunomodulatory and anti-inflammatory role in the other chronic inflammatory diseases such as IPF.

Animal models provide opportunities to explore the efficacy of potential therapeutic strategies. For example, various murine models recapitulate the features of IPF induced by diverse agents and different administration routes. A single intratracheal dose of bleomycin is a simple, popular and appropriate way to provide the well-characterized experimental IPF model to test the novel anti-fibrotic drugs [21]. Therefore, we used this animal model in this experiment. The current study was designed to evaluate the anti-fibrotic effect of PL in the treatment of IPF in vivo and demonstrate the possible underlying mechanisms.

## Materials and methods

### Animal and experimental model

Six- to eight-week-old C57BL/6 male mice (from the Animal Production facility of the Royan Institute, Tehran, Iran) were used in this experiment. Mice were kept in the standard cages at ambient humidity, room temperature, 12-h normal light-dark cycle, and fed freely. Animal experimentation was carried out with the approval of the Animal Care Committee of Iran University of Medical Sciences. All the animal works were approved by the ethics committee of the Iran University of Medical Sciences (IR.IUMS.FMC.REC1396.9511127007).

Pulmonary fibrosis was induced through non-surgical transoral instillation of bleomycin (Nippon Kayaku, Tokyo, Japan) intratracheally (IT) at the dose of 5 mg/kg in normal saline (at the final volume of 50 µl), to intraperitoneally (IP) ketamine/xylazine anesthetized mice. 2 mg/kg/day PL was administered IP for the first two weeks (day 1–14). The administered dose of PL was adapted from references [22, 23]. The non-surgical intratracheal instillation of bleomycin was performed as follows [24]. Briefly, the tongue of the mice was gently pulled out to one side using blunt forceps while the mouse was stabilized on the angled wooden platform hanging by its incisors on the wire and restrained gently with a ribbon. The trachea was visualized back of the mouth through a laryngoscope and 50 µl BLM or saline was inoculated into the trachea with the bent gavage needle.

Animals were assigned to the following groups: (i) Control group; which were treated IT with normal saline with the same volume of bleomycin solution (n = 10), (ii) Control + PL group; which were treated IT with normal saline and IP with Plumbagin (n = 10), (iii) BLM group; which were treated IT with bleomycin (n = 10), (iiii) BLM + PL mice; which were treated IT with bleomycin and IP with Plumbagin (n = 10). All animals were sacrificed at day 21 to further study.

### Histological analysis

Mice were sacrificed under deep anesthesia, and the lungs were isolated. The left lungs were fixed in 10% formalin for 24 h and embedded in paraffin. Paraffin-embedded tissue specimens were cut into 4 µm sections and stained with hematoxylin and eosin (H&E) and Masson’s trichrome according to standard histological procedures and observed blindly under a light microscope by an experienced pathologist.

### Hydroxyproline assay

The upper lobes of the right lungs were immediately snap-frozen and stored at − 80 °C and used to determine tissue hydroxyproline content. Hydroxyproline represents ~ 13.5% of the amino acid content of collagen and thus is a good indicator of the level of collagen present in the tissue samples [25]. A portion of the isolated lungs was weighed and acid-hydrolyzed overnight in 6 N HCl at 110 °C. Then hydroxyproline was measured with the Hydroxyproline assay kit (Sigma Aldrich, St. Louis, MO, USA) according to the manufacturer protocol. The hydroxyproline value per mg lung tissue was identified by comparing the absorbance of each sample to a standard curve generated by assay of known amounts of the standard concentrations.

### RNA isolation and real-time quantitative polymerase chain reaction (qRT-PCR)

The Frozen right lower lobes from the right lungs were cut into small fragments and homogenized in RNXPLUS reagent (Sinacolon) using a homogenizer. Total RNA of lung tissue homogenates isolated according to the manufacturer’s instructions (RNXPLUS reagent, Sinacolon). The cDNA was prepared by cDNA Synthesis Kit (TAKARA, Japan), and amplified using SYBR green reagents (TAKARA, Japan) in Rotor gene-Q PCR instrument with target genes primer sets CTGF (forward 5′-AGACCTGTGCCTGCCATTAC-3′, reverse 5′-ACGCCATGTCTCCGTACATC-3′), ET-1 (forward 5′-CTACGAAGGTTGGAGGCCAT-3′, reverse 5′-TGGGGGAGCTCTGTAGTCAA-3′), GUSB (forward 5′-GCTCGGGGCAAATTCCTTTC-3′, reverse 5′-CTGAGGTAGCACAATGCCCA-3′). Relative gene expression levels were evaluated using the 2^−ΔΔCt^ method and were normalized to the GUSB mRNA level and presented as fold change.

### Enzyme-linked immunosorbent assay

The levels of TGF-β1 and TNF-α cytokines were evaluated in tissue homogenates of the right lung, using Elisa kits (R&D Systems, Inc., MN, USA) according to the manufacturer’s protocol. Samples were prepared in duplicate.

### Statistical analysis

Comparative analysis among groups was made by the one-way ANOVA, followed by Tukey multiple comparison test running Prism software. P values < 0.05 were considered significant (*p < 0.05, **p < 0.01, ***p < 0.001). Data are displayed as means ± SEM.

## Result

### Plumbagin attenuated BLM-induced lung injury

To explore the potential protective effects of PL against lung fibrosis, histological characteristics of the lung tissue from each experimental group were determined by H&E staining and Masson’s trichrome staining of the tissue sections and by enzymatic measurement of the collagen content of isolated lung tissues. Since intratracheal bleomycin has been reported to cause fibrosis 14 days after administration, and the fibrotic response would peak through 21–28 days [26], histological changes were detected on day 21 in the group.

**Fig. 1:**
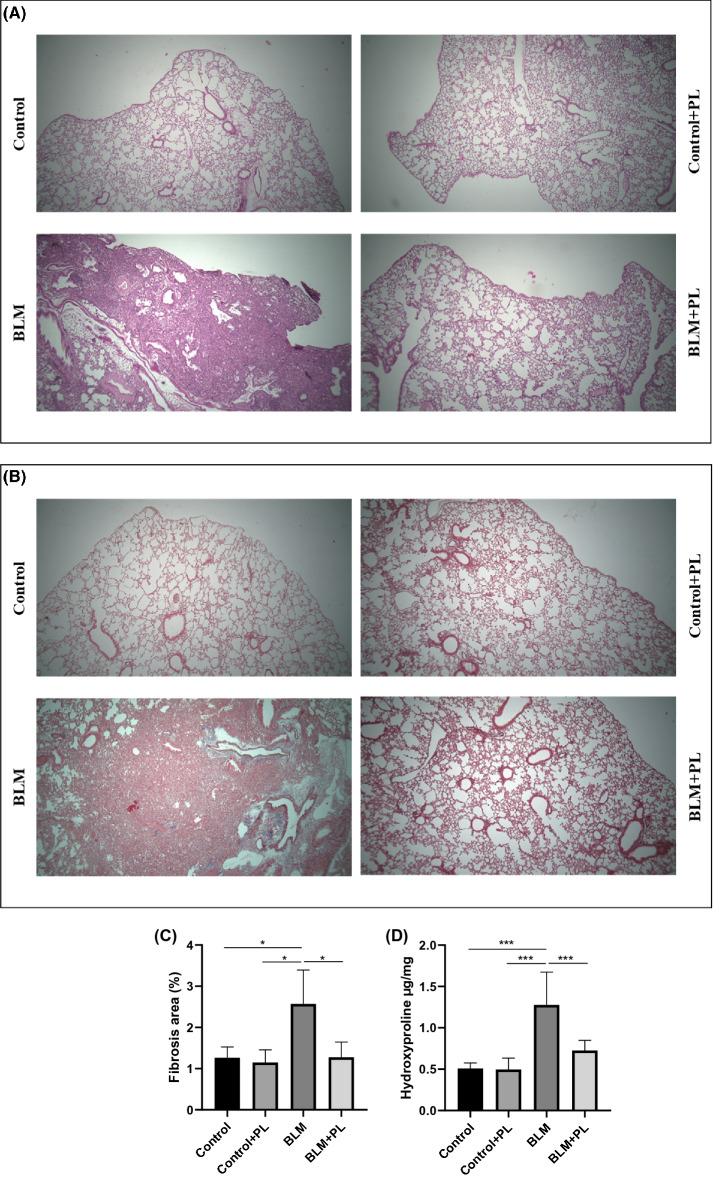
Examinations of fibrotic alterations in the mouse lungs from all of the experimental groups.** A** Histological examination of lung tissue was conducted by H&E staining, indicating the infiltration of immune cells and structural distortion. **B** Masson’s trichrome staining indicates the interstitial deposition of collagen (blue color) and the disruption of alveolar spaces. **C** Percent of the fibrotic area in the lung was quantitated by ImageJ software in trichrome staining sections. n = 3, in each group. **D** Evaluation of collagen contents was conducted by enzymatic assessment of hydroxyproline and values displayed per mg lung tissue. n = 10, in each group. Data are expressed as mean ± SD. ***p < 0.001, No drawing the comparative rectangle, and corresponding p-value means no significance

H&E-stained lung sections indicated that BLM challenge promoted inflammatory injury, and fibrotic alterations in the lung tissue were compared to saline treatment. Prominent interstitial inflammation and inflammatory cell infiltration were observed. Also, the normal structure of the lung was distorted with alveolar wall thickening and collapse of alveoli. While these changes were attenuated when BLM-received mice were treated with PL (Fig. 1A).

Further, Masson`s trichrome-stained lung sections showed a loss of alveolar architecture and increased collagen deposition extending, based on the increased intensity of blue color, in BLM challenged animals compared to control animals. Treatment with PL markedly reduced the BLM-induced collagen deposition in the interstitial area of lung tissue (Fig. 1B). Moreover, the percentage of the fibrotic area significantly increased in BLM group compared to the control (p < 0.05), which showed a significant decrease by PL treatment (p < 0.05) (Fig. 1C).

The measurement of hydroxyproline, considered the collagen tissue indicator, showed that its level was significantly increased in the BLM group compared to the control (1.277 vs. 0.5068 µg, p < 0.001). In line with morphological observation, the amount of hydroxyproline was significantly higher in BLM challenged mice compared to those treated with BLM and PL (1.277 vs. 0.7259 µg, p < 0.001), suggesting that PL diminished collagen deposition in the procedure of the BLM-induced lung fibrosis (Fig. 1D).

No significant differences in histological features and hydroxyproline content were observed among the control and control plus PL groups.

Collectively, the lung tissues obtained from the control and control plus PL group showed normal alveolar architecture without any fibrotic lesions, whereas the lung tissues from the BLM-induced model group displayed structural damage, interstitial inflammation, and fibrotic tissue with the collagen deposition. The above typical pathological features in the BLM-induced model group showed the successful modeling of lung fibrosis. Furthermore, after treatment with PL, the lung tissues recovered to the normal structure, and the fibrotic tissue and inflammation were reduced.

### Plumbagin inhibited the expression of CTGF and endothelin-1 (ET-1)

**Fig. 2:**
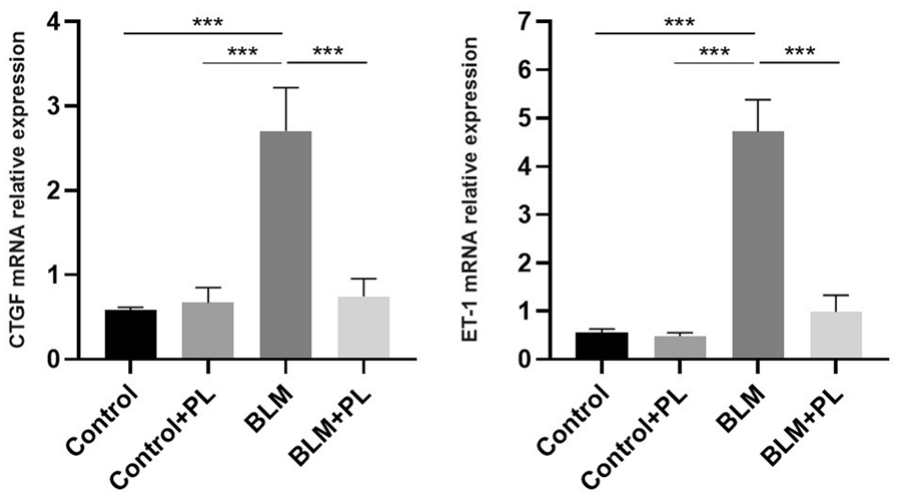
Effect of PL on expression level of CTGF and ET-1 in the mouse lung tissue from all of the experimental groups. The mRNA expression levels were normalized to the GUSB mRNA levels and displayed as fold change. n = 10, in each group. The values are represented as mean ± SD. ***p < 0.001, No drawing the comparative rectangle and corresponding p-value means no significance

To investigate the factors involved in fibrogenesis affected by PL, we analyzed the mRNA expression of CTGF and ET-1 in lung tissue isolated from each group and the result presented in Fig. 2 as fold changes. CTGF and ET-1 mRNA expression significantly increased in BLM-challenged animals compared with the control animals. However, a significant reduction of the CTGF and ET-1 expression was observed after PL treatment in BLM-received animals compared with those who received BLM only (p < 0.001). As we expected, no significant difference was observed in the expression of CTGF and ET-1 in control and PL –received mice (p > 0.5).

### Plumbagin suppressed TGF-β1 and TNF-α production

**Fig. 3:**
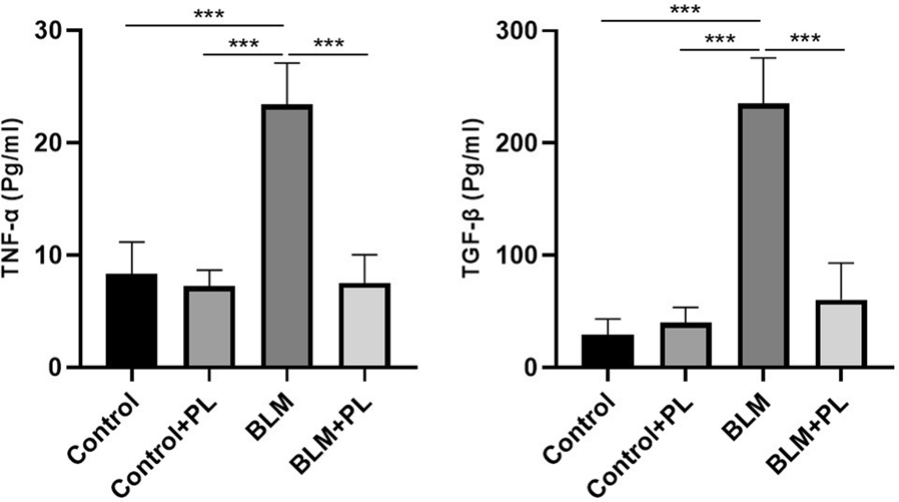
Effect of PL on TGF-β1 and TNF-α protein production expression levels from all of the experimental groups. The levels of cytokines were evaluated in tissue homogenates by ELISA assay. n = 10, in each group. The values are represented as mean ± SD. ***p < 0.001, No drawing the comparative rectangle, and corresponding p-value mean no significance

To further determine the factors mediating the suppressive effect of PL on IPF, we evaluate the level of two major cytokines that contributed in inflammation and lung fibrosis in the lung tissue of experimental groups (Fig. 3). The levels of TGF-β1 and TNF-α in lung tissue significantly increased in BLM challenged animals as compared with the control animals (p < 0.001). Whereas the levels of these two cytokines significantly decreased in BLM-received animals treated with PL, compared with the BLM group (p < 0.001). As we expected, the level of these cytokines was not different in control and control received PL mice (p > 0.5).

## Discussion

**Fig. 4:**
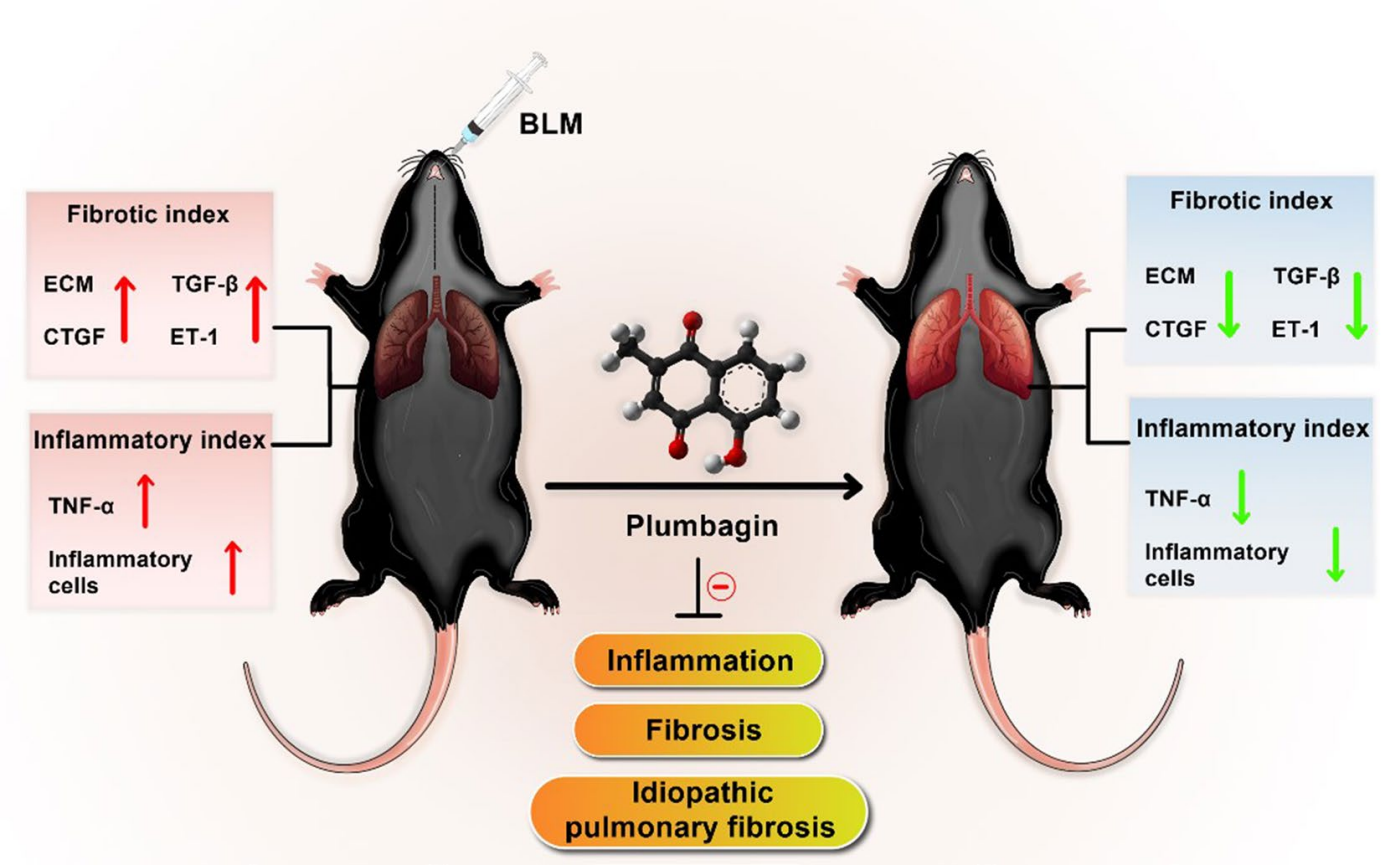
Graphic summary of anti-pulmonary fibrosis effect of Plumbagin in BLM-induced lung injury. PL can reverse pulmonary fibrosis by affecting both the inflammatory and fibrotic processes. *BLM* Bleomycin, *ECM* extracellular matrix, *TGF-β* tumor growth factor-beta, *CTGF* connective tissue growth factor, *ET-1* endothelin-1, *TNFα* tumor necrosis factor-alpha

Treatment of IPF remains a significant challenge due to the limited efficacy of current therapies [27]. PL, a naturally occurring compound, has been emerged as an anticancer agent, which acts against several aspects of tumorigenesis that also shared with fibrogenesis. PL has been proved to modulate several signaling pathways, such as Wnt, phosphatidylinositol 3-kinase (PI3K)/protein kinase B (Akt)/mammalian target of rapamycin (mTOR), AMP-activated protein kinase (AMPK), and epithelial–mesenchymal transition (EMT) [14]. Given the signaling pathways affected by PL and its immunomodulatory and anti-inflammatory functions, we speculated that this compound could be effective on IPF as a fibrotic disease provoked by a chronic inflammatory process. This investigation outlined the anti-fibrotic effect of PL in the experimental animal model of IPF. Results of present study revealed that administration of PL in a mouse model of lung fibrosis, effectively preserves lung structure, attenuates collagen deposition, reduces interstitial inflammation and inflammatory cell accumulation, as well as suppresses the crucial mediators involved in the fibrogenesis (Fig. 4).

Our present data indicated that exposure to BLM led to inflammatory infiltration, interstitial inflammation, over-expression of collagen, and architectural distortion of the lung that histologically resembles human IPF. The predominant cell type infiltrated into lung of the BLM-induced model were macrophages, followed by lymphocytes and neutrophils, and PL managed to eliminate BLM-induced pleiocytosis. PL administration displayed beneficial effect on all the IPF mentioned characteristics, suggesting the multiple activities of PL in improving the IPF. Our data are consistent with the previous findings reporting the effects of PL on reducing inflammation, diminishing collagen accumulation, preserving intact lobular architecture of liver tissue and improving liver fibrosis [17–20].

As the pro-fibrotic mediators, TNF-α and TGF-β play an essential role in alveolar damage, stimulation of fibroblast, and collagen deposition. TNF-α, mediate the inflammation and adaptive immune responses promoting fibrosis [28]. We showed that PL could significantly alter the BLM-induced up-regulation of TNF-α and TGF-β1. In support of our present data, blockade of TNF-α or TGF-β has been shown to diminish the inflammatory and consequent fibrotic response following BLM administration [29, 30]. In addition, previous studies demonstrated an inflammation-associated correlation between TNF-α and α-SMA, a marker of myofibroblast development [31], and PL decreased the expression of α-SMA and TNF-α and improved liver fibrosis [20].

TGF-β is the master regulator of fibrosis and is shown to be upregulated in the lung during fibrosis [32]. However, TGF-β1 is the most potent cytokine that contributes to the dysregulation of tissue repair mediating fibrosis, direct targeting of this pro-fibrotic mediator is anticipated to be problematic and has widely undesirable effects [33]. Two essential matricellular proteins, namely CTGF and ET-1, induced by TGF-β and act downstream of this cytokine to promote fibrogenic responses, including myofibroblast differentiation, collagen production, and α-SMA expression. CTGF and ET-1 have been shown to be upregulated in fibro-genesis and tumor-genesis processes [34–37]. A growing body of evidence indicated that TGF-β1, CTGF, and ET-1 expressions are cooperatively augmented within the lung tissue in IPF [38, 39], supporting that TGF-β/CTGF or ET-1 axis serves as the potential target for anti-fibrotic therapy. Our results showed that PL could reverse the expression levels of CTGF and ET-1 induced by BLM administration. Thus, PL causes a protective effect against lung fibrosis by both inhibiting TGF-β production and modulating the critical fibrogenic pathways downstream of TGF-β.

Briefly, in the pathogenesis of IPF, damaged alveolar epithelial cells promote the development of an actively pro-fibrotic environment by producing pro-fibrotic cytokines such as TGF- β, TNF-α, and CTGF and ET-1. In such an environment, the chronic activation of fibroblasts leads to continuously formation of myofibroblasts, collagen and fibrotic tissue [40]. Therefore, suppressing TNF-α, TGF- β, CTGF and ET-1 expressions involves the degradation of collagen and mitigating of lung fibrosis, which partially describes the anti-fibrotic mechanism of PL in IPF. However, how PL suppresses the expression of these factors is unclear. More recently, the therapeutic effect of PL in lung fibrosis has been linked to the inhibition of p300 histone acetyltransferase activity [41]. This study indicated that PL inhibited fibrotic target-gene expression and proliferation in response to TGF- β in the fibroblast cell line [41].

### Study limitation

Here we highlighted the effect of PL on several factors that contributed to lung fibrosis, including TNF, TGFb, CTGF or ET-1, but the precise underlying mechanism involved in this effect required further investigation. In addition, considering the wide range of mediators contributed in the development of fibrosis, it needs to take a more fibrotic factor into account for exploring anti-fibrotic effects of PL. It is also required to further validate the expression changes of all mentioned factors at both gene and protein levels for a more accurate conclusion.

## Conclusion

In conclusion, the data of this study suggest that PL can be a promising anti-fibrotic agent, which may be effective in preventing ECM deposition and thereby improve the IPF disease.

## Data Availability

The datasets used and/or analyzed during the current study are available from the corresponding author on reasonable request.

## Abbreviations

IPF: Idiopathic pulmonary fibrosis
PL: Plumbagin
H&E: Hematoxylin and Eosin
TGF-β: Tumor growth factor-beta
TNF-α: Tumor necrosis factor-alpha
CTGF: Connective tissue growth factor
ET-1: Endothelin-1
ECM: Extracellular matrix
EAE: Experimental autoimmune encephalomyelitis
EGFR: Epidermal growth factor receptor
IT: Intratracheall
EMT: Epithelial–mesenchymal transition
AMPK: AMP-activated protein kinase
BLM: Bleomycin
α-SMA: Alpha-smooth muscle actin
PI3K: Phosphatidylinositol 3-kinase
mTOR: Mammalian target of rapamycin

## References

[1] Zolak JS, de Andrade JA (2012). Idiopathic pulmonary fibrosis. Immunol Allergy Clin North Am.

[2] Wilson MS, Wynn TA (2009). Pulmonary fibrosis: pathogenesis, etiology and regulation. Mucosal Immunol.

[3] Selman M, King TE, Pardo A (2001). Idiopathic pulmonary fibrosis: prevailing and evolving hypotheses about its pathogenesis and implications for therapy. Ann Intern Med.

[4] Navaratnam V (2011). The rising incidence of idiopathic pulmonary fibrosis in the UK. Thorac Surg Clin.

[5] Raimundo K (2016). Clinical and economic burden of idiopathic pulmonary fibrosis: a retrospective cohort study. BMC Pulm Med.

[6] Raghu G (2012). Prednisone, azathioprine, and N-acetylcysteine for pulmonary fibrosis. N Engl J Med.

[7] Richeldi L (2014). Efficacy and safety of nintedanib in idiopathic pulmonary fibrosis. N Engl J Med.

[8] King TE (2014). A phase 3 trial of pirfenidone in patients with idiopathic pulmonary fibrosis. N Engl J Med.

[9] Richeldi L (2011). Efficacy of a tyrosine kinase inhibitor in idiopathic pulmonary fibrosis. N Engl J Med.

[10] Parimala R, Sachdanandam P (1993). Effect of Plumbagin on some glucose metabolising enzymes studied in rats in experimental hepatoma. Mol Cell Biochem.

[11] Xu TP (2013). Plumbagin from Plumbago Zeylanica L induces apoptosis in human non-small cell lung cancer cell lines through NF-κB inactivation. Asian Pac J Cancer Prev.

[12] Checker R (2009). Anti-inflammatory effects of plumbagin are mediated by inhibition of NF-kappaB activation in lymphocytes. Int Immunopharmacol.

[13] Wei Y (2017). Plumbagin restrains hepatocellular carcinoma angiogenesis by suppressing the migration and invasion of tumor-derived vascular endothelial cells. Oncotarget.

[14] Liu Y (2017). Anticancer Properties and Pharmaceutical Applications of Plumbagin: A Review. Am J Chin Med.

[15] Panichayupakaranant P, Ahmad MI (2016). Plumbagin and Its Role in Chronic Diseases. Adv Exp Med Biol.

[16] Zhang K (2014). Plumbagin suppresses dendritic cell functions and alleviates experimental autoimmune encephalomyelitis. J Neuroimmunol.

[17] Chen S, et al. Plumbagin ameliorates CCl4-induced hepatic fibrosis in rats via the epidermal growth factor receptor signaling pathway. Evid Based Complement Altern Med. 2015. 10.1155/2015/645727 PMC4624924 26550019

[18] Wang H (2016). Plumbagin protects liver against fulminant hepatic failure and chronic liver fibrosis via inhibiting inflammation and collagen production. Oncotarget.

[19] Chen Y (2019). Plumbagin ameliorates liver fibrosis via a ROS-mediated NF-кB signaling pathway in vitro and in vivo. Biomed Pharmacother.

[20] Wei Y (2015). Anti-fibrotic effect of plumbagin on CCl_4_-lesioned rats. Cell Physiol Biochem.

[21] Carrington R (2018). Use of animal models in IPF research. Pulm Pharmacol Ther.

[22] Yan W (2014). Plumbagin attenuates cancer cell growth and osteoclast formation in the bone microenvironment of mice. Acta Pharmacol Sin.

[23] Niu M (2015). Plumbagin inhibits growth of gliomas in vivo via suppression of FOXM1 expression. J Pharmacol Sci.

[24] Rayamajhi M, et al. Non-surgical intratracheal instillation of mice with analysis of lungs and lung draining lymph nodes by flow cytometry. J Vis Exp. 2011(51). 10.3791/2702 PMC3280633 21587154

[25] Stegemann H, Stalder K (1967). Determination of hydroxyproline. Clin Chim Acta.

[26] Moore B (2013). Animal models of fibrotic lung disease. Am J Respir Cell Mol Biol.

[27] Dempsey OJ (2006). Clinical review: idiopathic pulmonary fibrosis—past, present and future. Respir Med.

[28] Li X (2017). Drugs and targets in fibrosis. Front Pharmacol.

[29] Piguet PF (1989). Tumor necrosis factor/cachectin plays a key role in bleomycin-induced pneumopathy and fibrosis. J Exp Med.

[30] Giri SN, Hyde DM, Hollinger MA (1993). Effect of antibody to transforming growth factor beta on bleomycin induced accumulation of lung collagen in mice. Thorac Surg Clin.

[31] Palanisamy N, Kannappan S, Anuradha CV (2011). Genistein modulates NF-κB-associated renal inflammation, fibrosis and podocyte abnormalities in fructose-fed rats. Eur J Pharmacol.

[32] Lasky JA, Brody AR (2000). Interstitial fibrosis and growth factors. Environ Health Perspect.

[33] Mahendran S, Sethi T (2012). Treatments in idiopathic pulmonary fibrosis: time for a more targeted approach?. QJM.

[34] Leask A (2011). Possible strategies for anti-fibrotic drug intervention in scleroderma. J Cell Commun Signal.

[35] Zhu B (2013). Atorvastatin attenuates bleomycin-induced pulmonary fibrosis via suppressing iNOS expression and the CTGF (CCN2)/ERK signaling pathway. Int J Mol Sci.

[36] Grant K, Loizidou M, Taylor I (2003). Endothelin-1: a multifunctional molecule in cancer. Br J Cancer.

[37] Jacobson A, Cunningham JL. Connective tissue growth factor in tumor pathogenesis. In Fibrogenesis & tissue repair. BioMed Central. 2012. 10.1186/1755-1536-5-S1-S8 PMC3368788 23259759

[38] Tzortzaki EG (2007). Effects of antifibrotic agents on TGF-beta1, CTGF and IFN-gamma expression in patients with idiopathic pulmonary fibrosis. Respir Med.

[39] Abraham D (2008). Role of endothelin in lung fibrosis. Eur Respir Rev.

[40] Selman M, Pardo A (2003). The epithelial/fibroblastic pathway in the pathogenesis of idiopathic pulmonary fibrosis. Am J Respir Cell Mol Biol.

[41] Lee SY (2020). Plumbagin suppresses pulmonary fibrosis via inhibition of p300 histone acetyltransferase activity. J Med Food.

